# Computational Molecular Docking and X-ray Crystallographic Studies of Catechins in New Drug Design Strategies

**DOI:** 10.3390/molecules23082020

**Published:** 2018-08-13

**Authors:** Shogo Nakano, Shin-ichi Megro, Tadashi Hase, Takuji Suzuki, Mamoru Isemura, Yoriyuki Nakamura, Sohei Ito

**Affiliations:** 1 School of Food and Nutritional Sciences, Shizuoka University, Yada, Shizuoka 422-8526, Japan; snakano@u-shizuoka-ken.ac.jp (S.N.); yori.naka222@u-shizuoka-ken.ac.jp (Y.N.); itosohei@u-shizuoka-ken.ac.jp (S.I.); 2 Biological Science Research, Kao Corporation, Ichikai-machi, Haga-gun, Tochigi 321-3497, Japan; meguro.shinichi@kao.com; 3 Research and Development, Core Technology, Kao Corporation, Sumida, Tokyo 131-8501, Japan; hase.tadashi@kao.com; 4 Faculty of Education, Art and Science, Yamagata University, Yamagata 990-8560, Japan; taksuzuk@e.yamagata-u.ac.jp

**Keywords:** green tea catechins, EGCG, X-ray crystallographic analysis, computational molecular docking analysis

## Abstract

Epidemiological and laboratory studies have shown that green tea and green tea catechins exert beneficial effects on a variety of diseases, including cancer, metabolic syndrome, infectious diseases, and neurodegenerative diseases. In most cases, (−)-epigallocatechin gallate (EGCG) has been shown to play a central role in these effects by green tea. Catechins from other plant sources have also shown health benefits. Many studies have revealed that the binding of EGCG and other catechins to proteins is involved in its action mechanism. Computational docking analysis (CMDA) and X-ray crystallographic analysis (XCA) have provided detailed information on catechin-protein interactions. Several of these studies have revealed that the galloyl moiety anchors it to the cleft of proteins through interactions with its hydroxyl groups, explaining the higher activity of galloylated catechins such as EGCG and epicatechin gallate than non-galloylated catechins. In this paper, we review the results of CMDA and XCA of EGCG and other plant catechins to understand catechin-protein interactions with the expectation of developing new drugs with health-promoting properties.

## 1. Introduction

**Figure 1:**
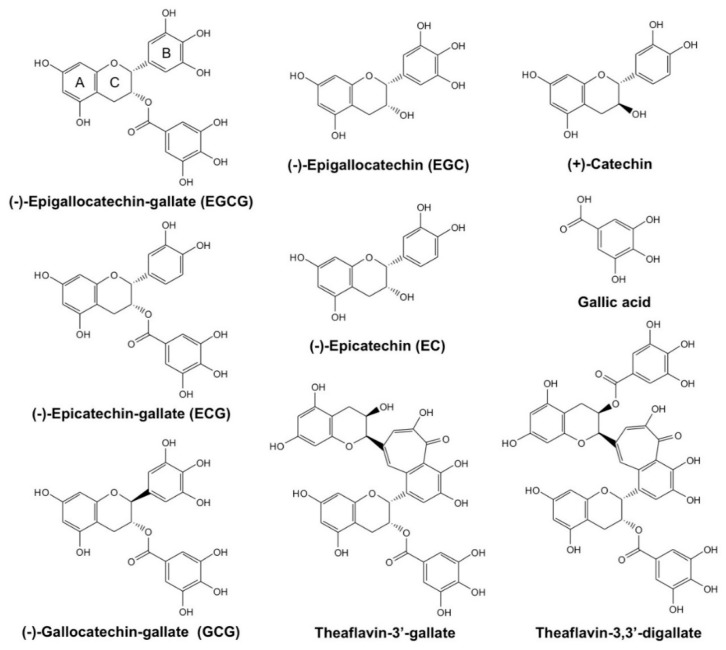
Chemical structures of catechin-related compounds.

Green tea and green tea catechins (GTCs) have been revealed to have beneficial effects on a variety of diseases such as cancer, metabolic syndrome (MetS), infectious diseases, and neurodegenerative diseases [1,2,3,4,5,6,7,8]. In most cases, (−)-epigallocatechin gallate (EGCG) is believed to play a central role in the biological actions of green tea. Plant catechins from other sources also show health benefits [8]. EGCG and other catechins contribute to these effects mainly through their antioxidative and prooxidative properties [1,2,3,4,5,6,7] (Figure 1). In addition, many studies have revealed that the binding of EGCG and other catechins to proteins is involved in its action mechanism [1,2,3,4,5,6]. Among studies on catechin-protein interactions, Arora et al. (1989) appears to be the first to provide detailed information on the interaction of serum albumin (SA) with catechins, determined by physico-chemical methods [9]. There are several methods to demonstrate catechin-protein binding interaction, including equilibrium dialysis, pH-metric and spectrophotometric methods, affinity chromatography, surface plasmon resonance, nuclear magnetic resonance, X-ray crystallographic analysis (XCA), and computational molecular docking analysis (CMDA) [5].

**Figure 2:**
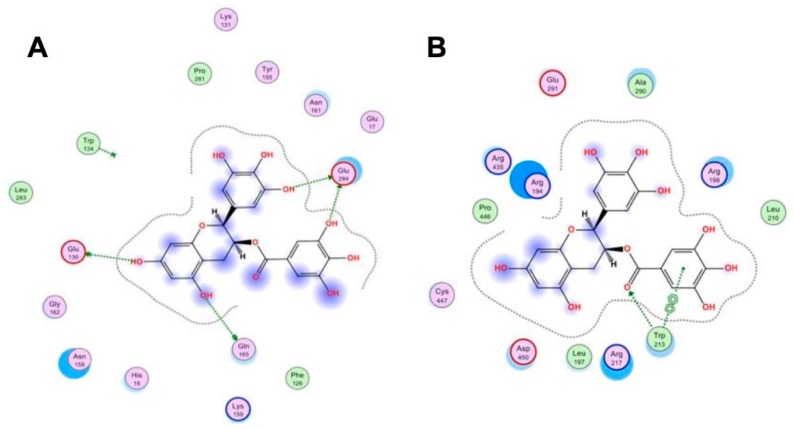
CMDA of EGCG binding to bovine SA. Interaction of EGCG with Trp134 (**A**) and Trp213 (**B**) are shown. Green broken arrows represent hydrogen bonding. The gallate group forms a hydrophobic bond with Trp213 (**B**). Cited from [12] under the terms of the Creative Commons Attribution-NonCommercial-ShareAlike License.

CMDA has been used to reveal further intricacies of the interaction between SA and catechins [5]. For example, data obtained by experiments that included CMDA indicated that EGCG binds to residues located in subdomains IIa and IIIa of human SA and that specific interactions occur with residues Trp214, Arg218, Gln221, Asn295, and Asp451 [10]. EGCG was shown to bind to IIa and IIIa domains of bovine SA, inducing a binding-dependent conformational change in the protein [11]. More recently, Ikeda et al. demonstrated that a gallate ester moiety at the C-3 position enhanced the binding ability of catechins to bovine SA by interacting with both Trp134 and Trp213 via π-π stacking (Figure 2). EGCG was also shown to interact with Glu130, Glu165, and Glu284, located on the surface of BSA, through hydrogen bonds [12]. Treatment of human hepatoma HepG2 cells with EGCG induced non-apoptotic cell death when serum proteins such as in fetal bovine SA and bovine SA were absent, and the toxicity of EGCG caused by its binding to various cellular proteins such as caspase-3, poly (ADP-ribose) polymerase, and α-tubulin, was reduced by the addition of SA [13]. CMDA indicated that bovine SA had a stronger affinity to EGCG than other proteins and that SA may act as a protein carrier. Binding information may be useful for developing a drug delivery system.

Shi et al. applied small-angle X-ray scattering to study the complex structure of bovine SA bound by catechin or EGCG [14]. Their results indicated that EGCG has a stronger ability than catechin to promote complex formation and further aggregation. The aggregates of EGCG complexes have a denser core with a relatively smooth surface, whereas catechin aggregates are loosely packed with a rough surface, suggesting a role of the gallate moiety in aggregate formation. Their findings provide a basic understanding of catechin-induced formation and aggregation of protein complexes [14].

In our previous review, we discussed the catechin-protein interaction as part of the mechanism of catechin’s beneficial actions in health and noted the importance of CMDA and XCA in elucidating the action mechanism of catechins [5]. In the present review, we expanded our literature search to review the results of analyses of EGCG and other catechin-related compounds (Figure 1) to further understand catechin-protein interactions with the expectation of developing new drugs with health-promoting properties.

## 2. CMDA of Catechin-Protein Interaction

### 2.1. Interaction of Catechins and Cancer-Related Proteins

Plant polyphenols have anticancer properties [1,2,3,4,5,6,7,8]. Catechins, especially EGCG, are the most extensively studied plant polyphenols. Several epidemiological studies showed the beneficial effects of green tea and GTCs on cancer [1,2,3,4,5,6,7]. The molecular binding interaction between GTCs and specific proteins is involved in tea’s anticancer mechanisms and CMDA was used to reveal such an interaction [5].

MMPs of cancer cells are believed to be involved in cell proliferation, invasion, and metastasis. For example, Bu et al. reported that the synthetic MMP inhibitor Batimastat exhibited inhibitory effects on tumor growth, tissue invasion, and intrahepatic and lung metastasis [15]. In the nude mice model of liver cancer, Batimastat prolonged survival of the animals [15]. EGCG and ECG have been shown to inhibit the enzymatic activity of MMPs, and GTCs and green tea have a proven record of inhibiting cancer metastasis in several animal experiments [16,17,18]. As discussed previously [5], CMDA demonstrated a binding interaction between GTCs and MMPs including MMP-2, MMP-9, and MMP-14, and the gallate group is important for establishing a strong interaction between MMP-2 and EGCG or ECG [19,20]. MMP-9 activity was inhibited by EGCG and ECG, but not by EC and EGC [20,21]. Their results showed interactions between EGCG and several amino acid residues in MMP-9 (Leu187, Leu188, Ala191, Glu402 (active site residue), His405, and Pro421) with an interaction energy exceeding 2 kcal/mol resulting from chemical bonds such as hydrogen bonds, π-π, π-cation, and π-σ interactions. These residues were also shown to be involved in the interaction with EC, EGC, and ECG. A galloyl moiety in EGCG interacts with amino acid residues such as Phe201, His401, Glu402, His 411, and Pro421. Due to the presence of a galloyl residue, EGCG and ECG had about 1.5-fold higher binding affinity with MMP-9 than EC and EGC, indicating its important role in the inhibitory action against MMP-9 activity [20].

**Figure 3:**
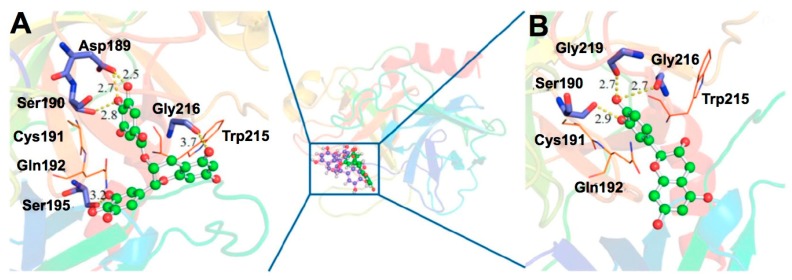
CMDA of complexes of trypsin and catechins. Representative complex structures for trypsine complex with EGCG (**A**) and catechin (**B**). EGCG and catechin are shown as ball-and-stick and trypsine as cartoon. Data are cited from a literature of Shi et al. [14]. Adapted with permission of Copyright (2017) American Chemical Society.

In the case of trypsin, which may be associated with the proliferation, invasion, and metastasis of cancer cells through degradation of the extracellular matrix proteins [22], Shi et al. suggested that binding of EGCG to the trypsin’s catalytic cavity involves hydrogen bonding provided by the hydroxyl groups in the galloyl residue, whereas that of catechin uses those in the B-ring [14] (Figure 3). Main contributors to hydrogen bond binding to EGCG were shown to be Asp189, Ser190, Ser195, Gly216, and Gly219, while those in the binding to catechin are Asp189, Ser190, Gly216, and Gly219. EGCG exhibited much stronger inhibitory activity than catechin. The data are consistent with those of Cui et al. who showed that in trypsin-catechin complexes residues Asp189, Ser190, Gln192, Ser195 and Val213-Ser214-Trp215 in common contribute to conserved hydrogen bond interactions or hydrophobic contacts [23]. Thus, catechin’s occupancy of the catalytic pocket of trypsin would hinder the substrate binding, leading to inhibition of the protein-degrading activity and the finding provides explanation of higher efficiency of galloylated catechins than non-galloylated catechins.

CMDA also suggested that a particular pose of EGCG may lead to covalent modification of the *N*-terminal threonine (Thr1) of the proteasome β5 subunit in the chymotrypsin-like active site [24]. Methylation of EGCG may reduce the biological activity of EGCG such as proteasomal chymotrypsin-like inhibitory activity. Methylation can disrupt the ability of ECG and EGCG to interact with Thr1 of this subunit. CMDA showed that methylation causes the ester carbon in these catechins to move away or be blocked entirely from Thr1, impairing the ability of these catechins to dock well. Their finding suggests that methylation inhibits the binding of these catechins to the proteasome β5 subunit, which would reduce their anticancer activity [24].

**Figure 4:**
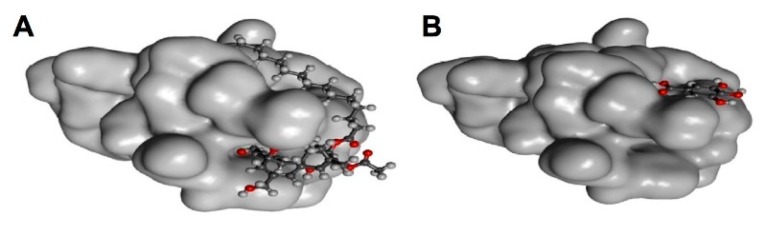
MCDA of binding interactions of PKCδ. Phorbol ester (**A**) and gallic acid (**B**) bind to the similar sites in PKCδ. Reproduced from [25] under the terms of the Creative Commons Attribution-NonCommercial-ShareAlike License.

Kongpichitchoke et al. examined the effect of gallic acid (Figure 1) on protein kinase C (PKC)δ [25]. They showed that the content of gallic acid increased depending on the degree of fermentation of tea and that gallic acid reduced reactive oxygen species (ROS) in phorbol ester-activated macrophages. Immunoblotting and CMDA revealed that gallic acid was able to block phosphorylation of PKCδ by occupying the phorbol ester binding sites of the protein (Figure 4). Thus, gallic acid at certain concentrations may protect cell death. Previously, in the 67-kD laminin receptor pathway, EGCG was shown to induce upregulation of cGMP which initiates cancer cell-specific cell death by activating PKCδ/acid sphingomyelinase [26], indicating the involvement of PKCδ activation in apoptosis. Thus, future study will be needed to clarify the effect of gallic acid on EGCG-mediated apoptosis in cancer cells.

Dutta et al. synthesized novel catechin/EC-based conjugates with resorcinol and phloroglucinol to increase the number of phenolic OH groups and found that the conjugates were more effective inhibitors of ribonuclease A than parental catechins, indicating the importance of number of phenolic hydroxyl groups on the inhibition of ribonucleolytic activity. CMDA revealed the precise protein-catechin interactions. The derivatives of EC, compounds **1** and **2**, also showed inhibited angiogenin-induced angiogenesis [27]. Since angiogenesis is associated with tumor growth, these catechin conjugates may have a potential role as anticancer agents.

To find chemotherapeutic agents, Shin et al. examined various plant-derived compounds including chalcones, which show the poor interaction with DNA and low risk of mutagenicity. Chromenones are also known to have anticancer effects and therefore, hybrids of chalcone and chromenone can be expected to be better chemotherapeutic agents. Shin et al. synthesized 16 chromenylchalcone compounds and screened their cytotoxicity against human colorectal cancer cell lines. Chromenylchalcone 11 showed a half-maximal inhibitory concentration (IC50) of 93.1 nM which is comparable with those of catechin gallate and ECG. The compound inhibited aurora kinases, and the mechanism could be examined by CMDA. These findings may provide useful information on synthesizing cancer therapeutic and preventive chromenylchalcone and catechin compounds [28].

**Figure 5:**
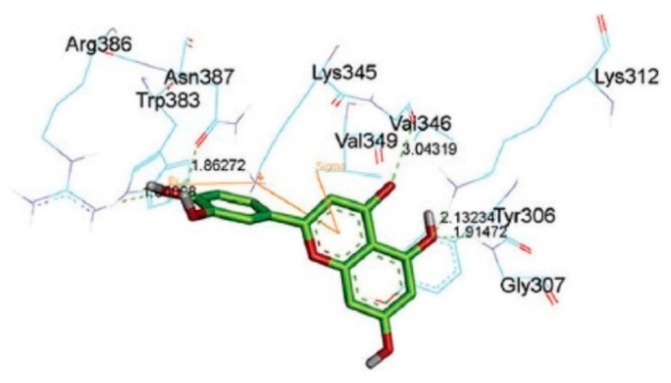
CMDA of a β-catenine-catechin complex. Green dotted lines and orange lines represent hydrogen bond and π interactions, respectively. Reproduced from [29] under the terms of the Creative Commons Attribution-NonCommercial-ShareAlike License.

Wnt signaling, which plays a crucial role in tumor cell proliferation, is correlated with the accumulation of β-catenin. Uncontrolled expression of β-catenin leads to fibromatosis, sarcoma, and mesenchymal tumor formation. Iftikhar et al. used CMDA and found that the binding energy of known inhibitors such as isorhamnetin, fisetin, genistein and silibinin is about −5 kcal/mol [29]. The corresponding value for catechin was also about −5 kcal/mol. The interaction of catechin and β-catenin involved six hydrogen bonds, two with β-catenin residue Lys312, and one each with Tyr306, Gly307, Lys345 and Asn387, respectively (Figure 5). Lys345 had a cation-π interaction with a ring, while hydrophobic interactions of the inhibitor were found with β-catenin residues Val346, Trp383, and Arg386. Flavonoid family members and T cell factor 4 competed for β-catenin binding by sharing common binding residues. These findings may be useful for screening β-catenin inhibitors as anticancer drugs [29].

Clinical studies have demonstrated a correlation between cyclin-dependent kinase 4 (CDK4) and malignant progression of pancreatic cancer. Phytochemicals such as wedelolactones and catechin were analyzed by several methods, including CMDA, to select anti-pancreatic cancer compounds. These compounds use less binding energy to CDK4 and inhibit its activity, suggesting them as potential drugs for treating pancreatic cancer [30].

Heat shock protein (Hsp)90 has served as a potential target in the therapy of breast and other cancers. Dunna et al. selected seven established Hsp inhibitors, EGCG, PU3, CCT-018159, CNF-2024, SNX-5422, AUY-922, and IPI-504. Considering these seven inhibitors as the parent compound, a ligand-based search was carried out, showing 90% similarity in the Pubchem database, which lists 31 million compounds. Among the compounds with the highest docking rerank scores, a new candidate (Pubchem CID: 11363378) that demonstrated considerable affinity towards Hsp90 was identified. Their study can serve as a model for searching for anticancer drugs based on the protein-ligand interaction [31].

Using single-cell alkaline gel electrophoresis (comet assay), Farhan et al. found the relative efficiency of cellular DNA breakage in the order of EGCG > EGC > EC > catechin and showed that DNA breakage was caused by mobilization of chromatin-bound copper ions and the generation of ROS [32]. The order of DNA binding affinity was confirmed by CMDA and a thermodynamic study using calf thymus DNA. Their results suggest that the synthesis of any novel anticancer molecule should have as many galloyl moieties as possible, which would provide an increased number of hydroxyl groups, which may facilitate the binding of the molecule to cellular DNA.

Chen et al. predicted that a serine/threonine kinase, 90-kDa ribosomal S6 kinase (RSK2), would be a molecular target of EGCG [33]. CMDA, when combined with a pull-down assay, indicated that EGCG binds to RSK2 at catalytic domains in vitro and ex vivo. Since RSK inhibition resulted in decreased cell migration and proliferation of cancer cells [34], EGCG may be useful in the prevention of cancer.

Cytosolic sulfotransferases (SULTs) are phase II detoxification enzymes that regulate the activity of more than hundreds of signaling small molecules with an allosteric effect. Genetic variation in *SULT1A1* is associated with the risk of certain types of cancer including lung cancer [35]. Using the spin-label triangulation method and CMDA, Cook et al. examined the structure of the catechin allosteric-binding site of SULT-1A1 and found that EGCG could control the opening and closure states of SULT-1A1 [36]. Their findings may be useful to understand allosteric regulation by EGCG and other compounds, and develop a drug that can enhance or diminish SULT activity.

Hepatic sinusoidal obstruction syndrome (HSOS) is a rare and life-threatening liver disease. Jing et al. investigated the involvement of the nuclear factor erythroid 2-related factor 2 (Nrf2) antioxidant signaling pathway in the protection of (+)-catechin against monocrotaline (MCT)-induced HSOS [37]. (+)-Catechin protected hepatic obstruction against MCT-induced HSOS in rats, attenuated the formation of ROS in human hepatic sinusoidal endothelial cells, enhanced Nrf2 nuclear translocation in livers from monocrotaline-treated rats and in MTC-treated cells, and further increased the expression of Nrf2-dependent genes. CMDA indicated the potential interaction of (+)-catechin with the Nrf2 binding site in the protein kelch-like ECH-associated protein-1 (Keap1), demonstrating the critical involvement of the Nrf2 pathway in protection by (+)-catechin against MTC-induced HSOS.

In a study that assessed the inhibitory effect of aqueous and methanolic extracts of 57 plants used in traditional Chinese medicine against the phase I metabolizing enzyme CYP3A4, the aqueous extract of *Acacia catechu*, which contains catechin, EC, and ECG, inhibited more than 85% of CYP3A4 activity when applied at a dose of 100 μg/mL. CMDA of major compounds showed that hesperidin and rutin had the highest fitting scores in the active sites of CYP3A4 with binding energy of about −74 and −71 kcal/mol, respectively. The corresponding value of catechin was about −39 kcal/mol, showing that it is a very weak inhibitor. These CYP3A4-inhibiting compounds may interfere with the metabolism of other concomitantly administered drugs [38].

Flavonoids have been found to possess anticancer activities by modulating different enzymes and receptors such as CDK6. Zhang et al. examined the binding of flavonoids which inhibited active CDK6 using CMDA and other methods. Their results indicated that among various flavonoids, six inhibitors—chrysin, fisetin, galangin, genistein, quercetin, and kaempferol—showed strong activity, with a binding energy in a range from −11 to −10 kcal/mol. The corresponding values of catechin and EC were greater than −9 kcal/mol, indicating that these catechins are weak inhibitors. When assessing the binding efficiencies of flavonoids against CDK6/cyclin D, the 3′-OH and 4′-OH of the B-ring favored hydrogen bond formation, whereas the 3-OH on the C-ring and 5-OH on the A-ring did not. The hydrogen-bonding force and van der Waals interactions with residues Ile19, Val27, Ala41, Glu61, Phe98, Gln103, Aap163, and Leu152 contributed to the binding affinity. These binding features of flavonoid inhibitors may provide valuable insight into the development of CDK6 inhibitors as chemopreventive agents [39].

**Figure 6:**
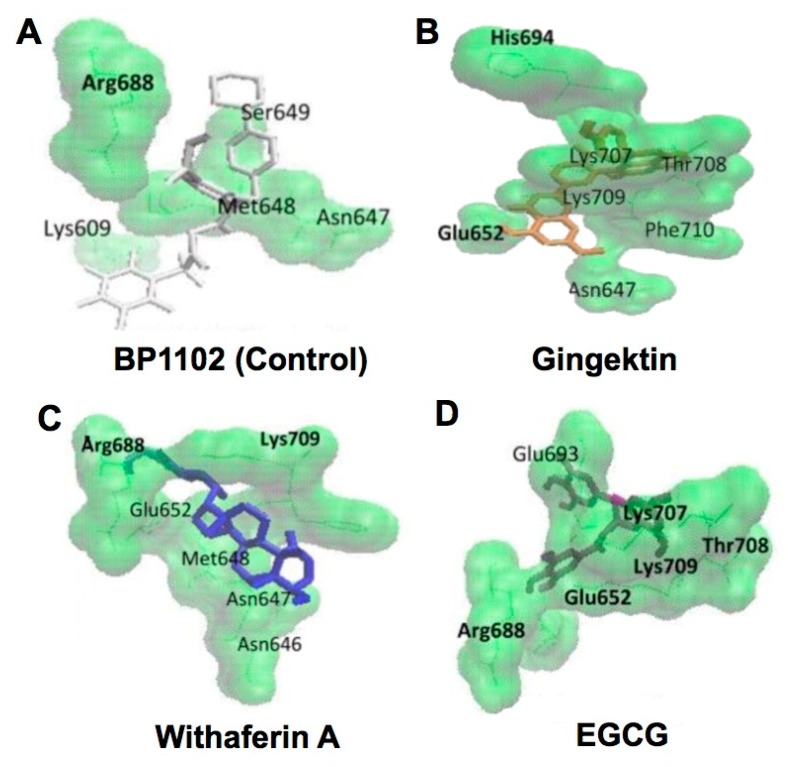
CMDA of binding interactions of selected compounds including EGCG with the DH2 domain of STAT3. (**A**) BP1102; (**B**) gingektin; (**C**) withaferin A; (**D**) EGCG. The amino acids with hydrogen bond interactions are shown in bold. Reproduced from [40] under a Creative Commons Attribution 3.0 License. PII: 17,466.

Transducers and activators of transcription 3 (STAT3) overexpression have been reported in many tumor types, suggesting that STAT3 inhibition can have an anticancer effect. Efferth et al. used the known STAT3 inhibitor BP-1-102 as a reference compound to study several potential anti-STAT3 compounds by methods including CMDA. Among 22 compounds assessed, six compounds including EGCG had a binding energy lower than that of BP-1-102 (around −7 kcal/mol), suggesting that they are more potent STAT3 inhibitors. The pKi values were 3.81 ± 1.94, 0.20 ± 0.06, 0.31 ± 0.07, 0.65 ± 0.27 for BP-1-102, gingektin, withaferin A, and EGCG, respectively, supporting that these three representative compounds are better inhibitor than BP-1-102. Amino acid residues involved in the binding interaction are: Lys609, Asn647, Met648, Ser649, Arg688 in BP-1-102; Asn647, Glu652, His694, Lys707, Thr708, Lys709, Phe710 in gingektin; Asn646, Asn647, Met648, Glu652, Arg688, Lys709 in withaferin A; and Glu652, Arg688, *Glu693*, Lys707, Thr708, Lys709 in EGCG (Figure 6). Thus, the amino acids participating in the EGCG-protein binding are also involved in the binding of at least one of the other three compounds, with the exception of *Glu693*. These amino acids involved in binding of the compounds to STAT3 are located in the Src homology 2 domain of the protein (Figure 6). The sample compounds provided in this study would be very useful for selecting and designing phytochemicals and drugs for cancer chemoprevention [40].

Sirtuin-6 (SIRT6) is an NAD^+^-dependent histone deacetylase that plays an important role in various biological events such as cancer, longevity, DNA-repair, and inflammation. Rahnast-Rilla et al. found that flavonoids can affect SIRT6 activity in a structure-dependent manner [41]. The most potent SIRT6 activator cyanidin produced a 55-fold increase in SIRT6 activity, while other flavonoids induced the 3–10-fold increase. Cyanidin also increased SIRT6 expression in human colorectal cancer Caco-2 cells. Binding of activators can change the conformation of a polypeptide loop near the acetylated peptide substrate binding site, leading to the activation of SIRT6. GCG and ECG showed significant inhibition against SIRT6 at 10  µM. CMDA predicted that the binding of the inhibitor can disturb the NAD^+^ binding to the enzyme. [41].

In addition to the above-mentioned studies, we have discussed the results of CMDA related to the interaction of EGCG with cancer-related proteins [5], including glucose regulated protein-78 [42], insulin-like growth factor-1 receptor [43], vascular endothelial growth factor (VEGF), VEGF1-receptor and VEGF2-receptor [44], zeta chain of T cell receptor associated protein kinase 70 [45], tyrosine kinases [46], tumor necrosis factor (TNF) receptor associated factor 6 (TRAF6) [47], urokinase [48], chymotrypsin [49], lipase [50], hydroxymethylglutaryl CoA reductase [51], leukotriene A4 hydrolase [52], DNA methyltransferase [53], phosphoinositide-3-kinase [54], protein phosphatases [55], and signal transducer and activator of transcription 3 (STAT3) [56].

### 2.2. Interaction between Catechins and Proteins Related to Neurodegenerative Diseases

Several epidemiological studies have shown that green tea and GTCs have beneficial effects on neurodegenerative disorders including Alzheimer’s disease (AD), Parkinson’s disease (PD), and cognitive dysfunctions [5]. For example, a meta-analysis of 26 observational studies showed that tea intake significantly reduced the risk of cognitive disorders (odds ratio = 0.65, 95% confidence interval = 0.58–0.73) [57]. A cross-sectional study on 1143 patients with a mean age of 68.9 in Japan found an association between low green tea consumption (p for trend = 0.032) with a higher prevalence of cognitive impairment [58]. Animal studies have supported these human results. For example, Unno et al. demonstrated that ingestion of GTCs suppressed brain dysfunction in aged mice [59].

Several investigations have revealed the molecular mechanism of catechin’s effects on neurodegenerative disorders using CMDA. In our previous paper, we reviewed the use of CMDA to assess the interaction between catechins and proteins related to neurodegenerative diseases [5,60,61,62,63,64]. Additional information is introduced next.

AD is the most common progressive neurodegenerative disorder, causing dementia, and involves the loss of structure and function of cholinergic neurons. Inhibitors of acetylcholinesterase (AChE) and butyrylcholinesterase (BChE) have potential in the treatment of AD. CMDA revealed that polyphenols inhibit the binding to AChE and BChE [65]. The binding energies of EGCG with AChE and BChE were lowest at about −14 and −13 kcal/mol, respectively. That study suggests that GTCs inhibit AChE and BChE, and enhance cholinergic neurotransmission by prolonging the transmission time. Since AChE molecules remain in the synaptic cleft, AChE inhibitors rather than BChE are suggested to be the standard drugs for the treatment of AD.

Similarly, Chen et al. examined AChE inhibitors present in plants available in markets. Using CMDA, the stability of the docked protein-ligand complex was assessed by molecular dynamics simulation [66]. The inhibitory assay demonstrated that *Uncaria rhynchophylla* and *Portulaca oleracea* extracts had AChE inhibitory activity. CMDA further indicated that catechin present in *U. rhynchophylla*, and dopamine and norepinephrine present in *P. oleracea*, had the best docking scores and interaction energy, suggesting that these compounds may be used to treat AD.

In a study that explored binding modes of catechins with molecular targets that have a potential role in PD, CMDA and other methods revealed monoamine oxidase-B as the most promising target, while *N*-methyl-d-aspartate receptor was the least favorable target for catechins [67]. A benzopyran skeleton with a phenyl group substituted at the 2-position and a hydroxyl or ester group at the 3-position were identified as common structural requirements in the majority of target proteins. These findings suggest that EGCG is the most promising lead to develop as a multitarget drug against PD.

Previous studies reported that EGCG can bind to unfolded native polypeptides and prevent conversion to amyloid fibrils. Amyloidogenic mutant chicken cystatin I66Q (CC166Q) is a generic amyloid-forming model protein that undergoes fibril formation through a domain-swapping mechanism. Wang et al. demonstrated that EGCG was a potent inhibitor of CC166Q fibril formation [68]. CMDA suggested that EGCG prevents fibril formation by stabilizing the molecule in its native-like state, as opposed to redirecting aggregation toward disordered and amorphous aggregates. That study suggests that EGCG is a generic inhibitor of amyloid-fibril formation, but the mechanism may differ from protein to protein. This finding may be useful to understand catechin’s inhibitory effect on protein fibril formation which can be a cause of certain forms of brain dysfunction.

Amyotrophic lateral sclerosis is a neurodegenerative disorder and mutations in the superoxide dismutase 1 (SOD1) protein contribute to rapid disease progression. Srinivasan and Rajasekaran examined the inhibitory action of EGCG against native and mutant SOD1, including by CMDA. Their results show that protein destabilization and increased β-sheet propensity in the mutant form were regained when bound by EGCG. The binding of EGCG to mutant SOD1 reduced the formation of toxic aggregates, suggesting a therapeutic potential for the treatment of this neurodegenerative disease [69].

**Figure 7:**
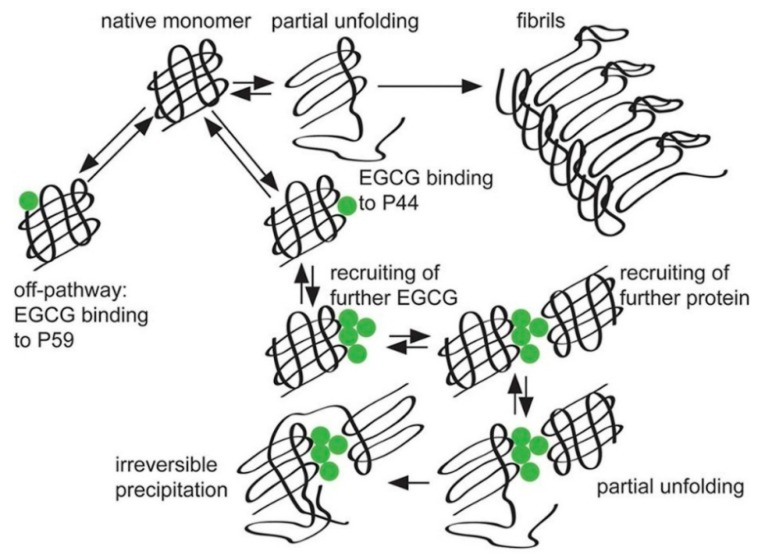
CMDA of the binding of EGCG with serum immunoglobulin light chains to cause fibril formation. A filled circle represents an EGCG molecule. Data are reproduced from Hora et al. [71] under a Creative Commons Attribution 4.0 International License.

Immunoglobulin light chain amyloidosis is a disease caused by fibril formation of serum immunoglobulin light chains and a common cause of acquired amyloid polyneuropathy [70]. EGCG is known to interfere with fibril formation by inducing protein aggregations. Hora et al. showed that NMR-guided CMDA identified two distinct EGCG binding sites, both of which include proline as a binding residue [71] (Figure 7). The EGCG-induced protein complexes are unstructured and EGCG reacts selectively with amyloidogenic mutants. Their proposed mechanism consists of three steps: (i) native monomers are converted via partially unfolded intermediates into amyloid fibrils, (ii) EGCG binds either to Pro59 (P59) with low affinity and no precipitation, or to Pro44 with higher affinity, and (iii) more EGCG molecules are recruited to the Pro44 (P44) binding site, which in turn results in the association of further proteins, leading to eventual precipitation (Figure 7). This makes EGCG a promising lead structure that is capable of handling the immense sequence variability of immunoglobulin light chains [71].

Down syndrome (DS) is a genetic disorder caused by the presence of all or part of a third copy of chromosome 21. The disease is associated with a decrease in the amount of mature nerve growth factor (NGF) by: (i) down-regulation of tissue plasminogen activator that activates plasminogen to plasmin, an enzyme that converts proNGF to mature NGF and (ii) overexpression of MMP-9, which degrades NGF. Wyganowska-Świątkowska et al. proposed an additional way in which EGCG might affect trisomy 21; namely, by modifying the proteolytic activity of the enzymes involved [72]. Although EGCG has previously been shown to inhibit urokinase [48] and docked well with it, it did not inhibit tissue plasminogen activator. EGCG was reported to inhibit MMP-9 [20,21] and docked well with MMP-9. Thus, EGCG may inhibit MMP-9 in the brain and slow the degradation of NGF, thereby preventing DS [72].

### 2.3. Interaction of Catechins and MetS-Related Proteins

The abnormal measures in MetS include those in body mass index, body fat, waist circumference, blood pressure, triglycerides, high-density lipoproteins, total cholesterol, blood glucose, and hemoglobin A1c. Patients with MetS have an elevated risk of diabetes mellitus and cardiovascular disease [1,4,5]. Several studies, including epidemiological studies, have shown that green tea and EGCG have beneficial effects against these diseases [1,4,5]. For example, a clinical trial found that a decrease in waist circumference of patients with diabetes who ingested catechin-rich green tea was significantly greater than that in patients who ingested green tea with much lower levels of catechins [73]. CMDA has revealed the mode of interaction of catechins with proteins related to MetS and related diseases, as exemplified by the studies examined next.

Rashid et al. reported that EC extracted from *Averrhoa carambola* L. peel was a bioactive compound that inhibits adipogenesis and obesity. CMDA suggested that EC appears to bind to some receptors, including CCAAT/enhancer-binding protein-α, peroxisome proliferator-activated receptor-α and γ [74].

The α-glucosidase inhibitor acarbose is a common oral anti-diabetic drug. Jhong et al. screened α-glucosidase and α-amylase inhibitors from 47 natural compounds using CMDA [75]. Three selected polyphenolic compounds (catechin, quercetin, and rutin) inhibited the activities of these enzymes when applied at less than 0.5 mM, which is comparable to the effectiveness of acarbose. Molleno-Ulloa et al. reported that the mode of binding of G-protein-coupled receptor to EC is similar to that to its agonist, G1 [76]. Wagner et al. attempted to reveal the molecular mechanism of how EGCG-rich green tea extract affects glucose metabolism and increases fitness and lifespan in the fruit fly, *Drosophila melanogaster* [77]. CMDA indicated that EGCG can inhibit glucose generation by binding to the active site of α-amylase, leading to a caloric restriction-like effect which would extend lifespan [78].

Sun et al. examined the interactions between pancreatic α-amylase (PPA) and tea polyphenols by biochemical and physicochemical methods such as CMDA, fluorescence quenching, and differential scanning calorimetry. They showed that the galloyl moiety interacts with PPA through hydrogen bonds between its three hydroxyl groups and the catalytic amino acid side-chains (Asp197, Glu233 and Asp300) and also through hydrophobic π-π interactions with the active site of the enzyme (Trp59). Tea polyphenols included EGCG, ECG, EGC, EC, theaflavin-3′-gallate, and theaflavin-3, 3′-digallate. The binding constants obtained through fluorescence quenching and isothermal titration calorimetry are very similar among these polyphenols, suggesting that their binding to PPA primarily includes an interaction with Try and with the vicinity of Try at the active sites of the enzyme. Their findings suggest that the galloyl moiety in these polyphenols binds to PPA and promotes polyphenol entering and association with the active site of the enzyme [79].

Flavonoids may reduce the risk of cardiovascular disease by inhibiting the activity of kinases such as phosphoinositide-3-kinase, Fyn, Lyn, Src, and PKC. XCA showed that flavonoid ring systems and their hydroxyl substitutions are important for the binding of flavonoids to kinases. A clearer understanding of structural interactions between flavonoids and kinases is necessary to allow the construction of more potent and selective counterparts. Wright et al. examined the interactions of quercetin, apigenin and catechin with Src family kinases (Lyn, Fyn and Hck) by several methods, including CMDA, and revealed potential hydrogen bond contacts between flavonoid hydroxyls and kinase catalytic site residues [80]. Quercetin formed the most energetically stable interactions, apigenin lacked the hydroxyl groups necessary for important contacts and the non-planar structure of catechin could not support predicted hydrogen bonding patterns. The findings by Wright et al. predict that quercetin would inhibit the activity of Src family kinases with greater potency than apigenin and catechin. This prediction was confirmed using in vitro kinase assays [80].

### 2.4. Interaction of Catechins with Proteins Related to Infectious Diseases

Several human studies have confirmed tea’s beneficial effects on infectious diseases. For example, in a case-control study, drinking green tea had a significant negative association with tuberculosis with an odds ratio of 0.534 [81]. Catechins have antibacterial activity and can modify the properties of multidrug-resistant *Staphylococcus aureus* (MRSA). Taylor comprehensively discussed the binding avidity of catechins, among which ECG showed the strongest binding to MRSA [82]. Incubation of MRSA with ECG inhibited bacterial growth and gave a dark color to MRSA, indicating that ECG bound to MRSA. Thus, catechin gallates are attractive agents for therapeutic intervention. In a clinical trial, Sameshima et al. demonstrated that green tea powder greatly potentiated the effect of interferon/rivabirin therapy in intractable chronic hepatitis C patients [83]. Song reviewed laboratory studies of antiviral activities of catechins on diverse families of viruses, including hepatitis B and C virus, human immunodeficiency virus, *Herpes simplex* virus, and influenza virus [84]. CMDA has provided a molecular basis to explain how catechins exert anti-microbial effects as shown next.

Sharma et al. showed that EGCG inhibited enoyl-acyl carrier protein reductase of *Mycobacterium tuberculosis*, with an IC50 of 17.4 μM by interfering with the binding of NADH to the enzyme [85]. A direct binding assay using [^3^H]EGCG and a fluorescence titration assay supported the inhibition data which were confirmed by CMDA. These findings may be useful, since this enzyme is a target for antitubercular drugs.

Universal stress protein is a novel target to overcome tuberculosis resistance. CMDA and other experiments showed that curcumin, catechin, and resveratrol have Arg136 hydrogen bonding and two ionic bonds between the carboxyl group of curcumin and Leu130 and Asn144, respectively [86]. Curcumin showed highest binding energy (about −9 kcal) with universal stress protein, while quercetin and catechin had the larger binding energy values. The 3, 4-dihydroxyphenyl ring in catechin participated in hydrogen bonding with the protein. Curcumin analogues may be useful for future therapy to down-regulate universal stress protein.

Gradisar et al. examined the inhibition of catechins against bacterial DNA gyrase B by binding to the ATP binding site and found that EGCG had the highest activity, followed by ECG and EGC [87]. Chemical shift perturbation was used to determine the protein moieties affected by binding to EGCG. CMDA suggested that the benzopyran ring of EGCG penetrates deeply into the active site, while the galloyl moiety anchors it to the cleft through interactions with its hydroxyl groups, explaining the higher activity of EGCG and ECG than EGC and EC. EGC was suggested to interact with gyrase mainly through interaction with its B-ring.

*Staphylococcal* enterotoxins are metabolic products of *Staphylococcus aureus* that are responsible for food poisoning. The results of fluorescence emission spectrometry and CMDA indicated that EGCG showed an order of magnitude higher binding constant (1.4 × 10^5^/ M) than other polyphenols studied. EGCG and similar polyphenolic ligands likely bind to the channel at the surface of *Staphylococcal* enterotoxin B, which is responsible for recognition of the T-cell β chain fragment and influence adhesion of the toxin to T cells [88].

**Figure 8:**
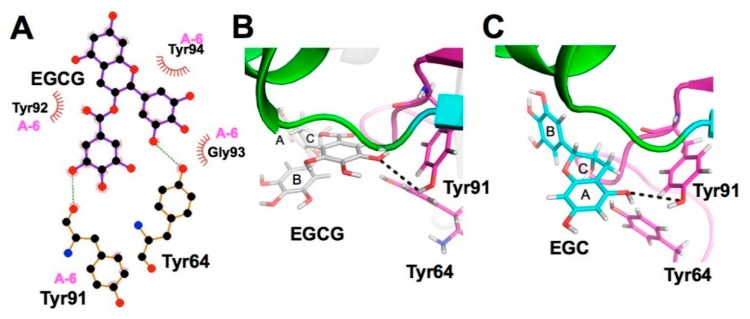
Autodock blind-docking of EGCG and EGC to SEA protein. Ligplot and docking simulation for the binding of EGCG to the active site of the A-6 region (**A**,**B**) and for the binding of EGC to the same region (**C**). The gallate moiety participates in the hydrogen bonding with Tyr 91 (**A**,**B**), while the A ring does so in EGC (**C**). Data are reproduced from Shimamura et al. [89] under the Creative Commons Attribution License.

Similarly, Shimamura et al. found that EGCG bound strongly to *Staphylococcal* enterotoxin A (SEA). The results derived from several methods including CMDA indicated that the hydroxyl group at position 3 of the galloyl group in EGCG was responsible for binding to Tyr91 in the A-6 region of the toxin’s active site [89] (Figure 8). Moreover, the hydroxyl group of the A ring of EGCG forms a hydrophobic bond with Tyr91 (Figure 8). Since 3″-*O*-methylation of EGCG resulted in a weaker binding interaction with the protein than EGCG itself, hydrogen bonding with Tyr91 of the 3″ hydroxyl group in the galloyl group must be important for efficient interaction, leading to the toxin’s activity. CMDA also predicted that EGC binds to the toxin’s A-6 region, similar to EGCG, but that the hydroxyl group in the A-ring is involved in binding to Tyr91 (Figure 8). Thus, the efficient inhibition of the toxin’s activity can be accounted for by involvement in the interaction of the galloyl group with SEA.

In a study of the mechanism underlying the antibacterial activity of EGCG against Gram-positive bacteria *Bacillus subtilis*, EGCG treatment resulted in formation of protein aggregates such as oligopeptide ABC transporter binding lipoprotein (OPPA), glucose phosphotransferase system transporter protein, phosphate ABC transporter substrate-binding protein, and penicillin-binding protein 5 [90]. EGCG inhibited the major functions of these proteins, leading to the inhibited growth of *B. subtilis*. For OPPA, CMDA showed that EGCG binds to basic amino acids located in the inner wall of OPPA through hydrogen bonding and forms a stable complex to prevent the conformational change of OPPA from an opened to a closed state, leading to the inhibited transport of oligopeptides.

**Figure 9:**
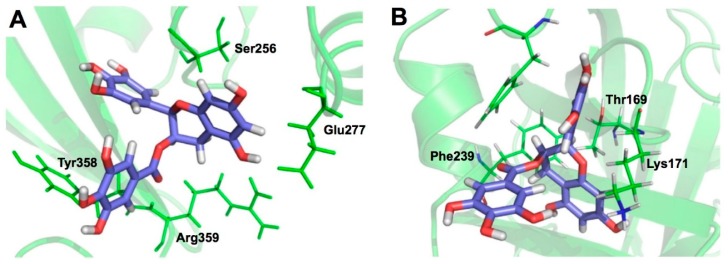
CMDA of the binding interaction of EGCG with PLY (**A**) and SrtA (**B**). CMDA predicts the involvement of Ser256, Glu277, Tyr358, and Arg359 in PLY and Thr167, Lys169, and Phe237 in SrtA in the binding to EGCG. Reproduced from [91] under the terms of the Creative Commons Attribution License (CC BY).

*Streptococcus pneumoniae* possesses numerous virulence factors associated with *pneumococcal* infection and pathogenesis. Pneumolysin (PLY) is a member of the cholesterol-dependent cytolysin family and has cytolytic activity. Sortase A (SrtA) contributes to the anchoring of many virulence-associated surface proteins to the cell wall. Song et al. showed that EGCG directly inhibited PLY mediated haemolysis and cytolysis by blocking the oligomerization of PLY and simultaneously reducing the peptidase activity of SrtA. EGCG also inhibited biofilm formation, the production of neuraminidase A, and bacterial adhesion to human epithelial Hep2 cells. CMDA and mutational analysis predicted that EGCG binds to Glu277, Tyr358, and Arg359 in PLY and Thr169, Lys171, and Phe239 in SrtA (Figure 9). Since EGCG protected mice against pneumonia by *S. pneumoniae*, EGCG may be a promising therapeutic option for this disease [91].

The binding gp120 of human immunodeficiency virus type 1 (HIV-1) to cell surface CD4 is the initial step of viral entry into cells. EGCG is an inhibitor which blocks gp120-CD4 binding. Hamza et al. examined the inhibitory mechanism by using CMDA and other methods to predict the most favorable structures of CD4–EGCG, gp120–CD4, and gp120–CD4–EGCG binding complexes in water. EGCG bound with CD4 in such a way that the calculated binding affinity of gp120 with the CD4–EGCG complex was negligible and the favorable binding of EGCG with CD4 effectively blocked gp120–CD4 binding [92]. It was suggested that EGCG can block CD4-gp120 binding, with an IC50 of about 100 μM. These findings would be useful for the development of next-generation anti-HIV-1 drugs.

3C-like protease (3CLP) of severe acute respiratory syndrome-associated coronaviruses plays a pivotal role in viral replication and is a promising drug target. Nguyen et al. used recombinant 3CLP to study inhibition and kinetics with seven plant flavonoids [93]. The IC50 values of these flavonoids ranged from 47 to 381 μM. Quercetin, EGCG, and gallocatechin gallate (GCG) were good inhibitors with IC50s of 73, 73 and 47 μM, respectively. GCG exhibited competitive inhibition with a pKi value of 25 ± 1.7 μM. CMDA showed that GCG with a binding energy of around −14 kcal/mol to the active site of 3CLP and the galloyl moiety at the 3-OH position was required for 3CLP inhibition activity [93].

Derksen et al. examined the anti-influenza A virus (IAV) potential of a proanthocyanidin-enriched extract of the aerial parts of *Rumex acetosa* (RA) [94]. The extracts inhibited the growth of IAV strain PR8 (H1N1) and a clinical isolate of IAV(H1N1)pdm09 with IC50s of 2.5 and 2.2 µg/mL, respectively. RA extracts were also active against an oseltamivir-resistant isolate. Procyanidin B2-digallate (epicatechin-3-*O*-gallate-(4β → 8)-epicatechin-3′-*O*-gallate, PBD) was identified as the main active principle of RA (IC50 was ca. 15 µM). RA and PBD blocked the attachment of IAV and interfered with viral penetration at higher concentrations. Galloylation of the procyanidin core structure was a prerequisite for anti-IAV activity; *O*-trihydroxylation in the B-ring increased anti-IAV activity. CMDA indicated that PBD interacts with the receptor binding site of IAV(H1N1)pdm09 hemagglutinin. RA and PBD appear to be a promising expansion of currently available anti-influenza agents [94].

Chikungunya fever is a viral disease that may cause chronic arthritis. Jain et al. evaluated protein-ligand interactions of all chikungunya virus proteins with natural compounds to identify potential inhibitors by methods including CMDA and found that catechin-5-*O*-gallate and rosmarinic acid interact with viral capsid protein. The top docking conformation of catechin-5-*O*-gallate showed a predicted binding energy of about −6 kcal/mol and rosmarinic acid had slightly larger binding energy. These compounds may serve as lead compounds to develop antiviral drugs [95].

Fatima et al. prepared HCV NS3 helicase from Pakistani HCV serum samples and conducted CMDA based on its 3D structure to identify inhibitors against HCV genotype 3a. Among various compounds, including quercetin, catechins, resveratrol, and lutein, the former two compounds showed good hydrogen bond interactions with best docking energy. These anti-helicase molecules would offer a clue in the development of antiviral drugs [96].

Additional CMDA studies on the interaction between catechins and cholera toxin, Dengue virus envelope glycoprotein E, and papillomavirus E6 oncoprotein [97,98,99] have already been discussed [5].

### 2.5. Interaction of Catechins with Proteins Related to Immunological Disorders

EGCG and catechin derivatives are suggested to have a role as preventive agents for immunological disorders such as food allergy and rheumatoid arthritis. Several human, cellular, and animal experiments have shown catechin’s beneficial effects on allergy and rheumatoid arthritis. For example, a randomized, double-blind, placebo-controlled trial showed that green tea containing *O*-methylated catechins reduces symptoms of Japanese cedar pollinosis [100]. In an animal model of arthritis, the therapeutic effect of EGCG was reported to be comparable to that of methotrexate, a well-known anti-rheumatoid arthritis drug [101]. CMDA provided information on the interaction of catechins with proteins related to immunological diseases and two studies [102,103] have been discussed earlier [5].

CMDA analysis showed that EGCG binds to the pocket that partly overlaps with the IgE-binding region in ovalbumin, a major dietary allergen, and that the interaction induces structural changes in ovalbumin. The ex vivo studies revealed that ovalbumin bound to IgE and stimulated degranulation of basophils and that EGCG slowed down its uptake by monocytes. Thus, EGCG might interact with food allergens and impair antigen uptake by antigen-presenting cells [104].

Peanut 2S albumins are seed storage proteins and major elicitors of anaphylactic reactions to peanuts. Using various methods including CMDA to elucidate interactions with EGCG, Vesic et al. demonstrated that similar structure and overall fold of 2S albumins yielded similar putative binding sites and similar binding modes with EGCG. Binding of EGCG to 2S albumins affected protein conformation by causing a transition from an α-helix to a β-structure, but the effect of EGCG on anaphylactic reactions remains unknown [105].

Xing et al. examined the anti-inflammatory effects of procyanidin B1, a (+)-catechin oligomer derivative, by biochemical methods and CMDA [106]. They identified Tyr296 in toll-like receptor 4 (TLR4) and Ser120 in myeloid differentiation factor 2 (MD-2) as critical sites for hydrogen bonding with procyanidin B1, similar to the sites occupied by LPS. The production of TNF-α was significantly decreased by procyanidin B1 in LPS-treated THP1 cells. Procyanidin B1 also significantly decreased phosphorylated p38 MAPK and NF-κB proteins, as well as mRNA levels of MD-2, TRAF-6, and NF-κB. Procyanidin B1 can compete with LPS for binding to the TLR4-MD-2 complex and suppress downstream activation of p38 MAPK and NF-κB signaling pathways.

These results collectively indicate that catechins effectively inhibit downstream inflammatory signaling. Non-covalent interactions and additional hydrogen bonds of EGCG and other catechin derivatives with proteins are likely to contribute to its diverse biological activities.

### 2.6. Miscellaneous

Many research groups have reported that tannins function as antioxidants and anticarcinogens using a combination of computational and biochemical assays. Shraberg et al. demonstrated that procyanidin tannin stereoisomers, such as procyanidin B1–B4, exhibit different binding affinities for the salivary peptide histatin 5 by electron spray ionization mass spectroscopy [107]. Computational analysis, such as CMDA and molecular dynamics simulation, suggested that tannin can make close contact with a variety of amino acid residues on the peptide.

The involvement of peroxidases and polyphenol oxidase (PPO) in the enzymatic browning reaction of fruits and vegetables with different catalytic mechanisms is well established. Both enzymes have some common substrates, but each also has specific substrates [108]. Nokthai et al. conducted a computational study on construct models employing the amino acid sequence of grape peroxidase and the homology modeling method based on the X-ray structure of ascorbate peroxidase from pea (Protein data base (PDB) ID:1APX) [108]. The model of grape PPO was obtained directly from the X-ray structure (PDB ID:2P3X) reported previously. CMDA of common substrates with those enzymes revealed that EC and catechin exhibited a high affinity to both enzymes, although peroxidase and PPO have binding pockets different both in the size and in key participant amino acids. The calculation of docking interaction energy for trihydroxybenzoic acid-related compounds led to the suggestion that these compounds have potentials as a common inhibitor against ascorbate peroxidase and PPO.

## 3. X-ray Crystallographic Analysis (XCA) of Protein-Catechin Complexes

Compared to CMDA, catechin-protein complexes have been studied less extensively by XCA. For example, PubMed reports only four original studies to date when searched with the keywords EGCG, protein, interaction, and X-ray. Generally, XCA provides more detailed information of molecular interaction with proteins and ligands than CMDA. In a previous paper, we reviewed XCA of EGCG-Pin 1, EGC-glutamate dehydrogenase (GDH), and EGCG-transthyretin complexes [5]. Here, we extend that discussion by using HOMOCOS, a modeling protein-compound complex structure server [109], to analyze the interaction between catechins and proteins. Ligand-protein interactions are defined by RCSB PDB Ligand Explorer ver. 4.2.0. Table 1 summarizes the findings for 10 catechin-protein complexes.

### 3.1. XCA of (+)-Catechin-and EC-Protein Complexes

**Figure 10:**
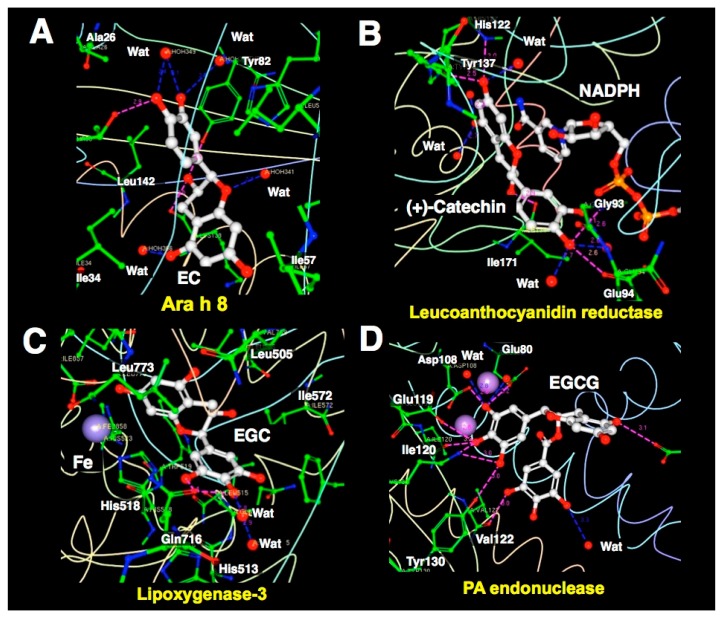
Representative interactions of EGCG or ECG with proteins as revealed by XCA. (**A**): Ara h 8 complex with EC. The residues that form hydrophobic interactions are not displayed. (**B**): Leucoanthocyanidin reductase complex with (+)-catechin and NADPH. The residues that form hydrophobic interactions are not displayed. The nicotinamide and the C ring of ECG stack in parallel. (**C**): Soybean lipoxygenase-3 complex with iron and ECG. The residues that form hydrophobic interactions are also displayed in a wire-frame model. Hydrophobic contact is defined by the number of atoms within 3.9 Å of the ligands. (**D**): PA endonuclease complex with EGCG. Extensive hydrogen bonds observed in PA endonuclease. The residues that form hydrophobic interactions are not displayed. The red dotted lines represent the observed hydrogen bonds (within 3.3 Å). The blue dotted lines represent bridged hydrogen bonds. Figures were prepared using RCSB PDB Ligand Explorer software.

Ara h 8, which is a peanut allergen, is thought to be responsible for oral allergy syndrome. Ara h 8 binds EC, and their complex was determined in 2.0 Å resolution [110]. XCA shows a graphical depiction of the interaction of EC with the protein (Figure 10A). Leucoanthocyanidin reductase is an enzyme that catalyzes the NADPH-dependent reduction of flavan-diols, a subfamily of flavonoids which may possess beneficial properties in human health. Mauge et al. conducted XCA of leucoanthocyanidin reductase from *Vitis vinifera* complexed with NADPH and (+)-catechin [111]. The result is shown in Figure 10B.

The structure of the ternary complex, determined at a resolution of 2.3 Å, indicates the π-π interactions between (+)-catechin and the nicotinamide ring of NADPH and suggests that His122 and Lys140 act as acid-base catalysts. This result indicates that catechins could bind to the active site and interact directly with the cofactor.

The strawberry allergen 10 Fra a proteins are involved in many aspects of plant biology and are related by sequence and structural homology to mammalian lipid transport receptor proteins. Casañal et al. showed that these proteins bind natural flavonoids with different selectivities and affinities [112]. XCA of Fra a-1E and Fra a-3 complexes with (+)-catechin indicates that several loops surrounding the ligand-binding cavity have significant flexibility in the apo forms, but would close upon ligand catechin binding. The A-ring is near the iron co-factor and attached by a hydrogen bond to the *C*-terminus of the enzyme, the B-ring hydroxyl groups participate in hydrogen bonds, and van der Waals interactions are formed by the surrounding amino acids and water molecules. It is likely that hydrogen bonding interaction between catechin and Fra a 1E was not observed because of their vigorous motion.

In addition, XCA shows several hydrogen bonds not only between catechins and proteins, but also between catechin and waters, which cannot be revealed by CMDA. The average numbers of hydrogen bonds and atoms that make hydrophobic contact with EC-waters and EC-proteins are 6.0 and 5.5, respectively, except for low-resolution Fra a 1E complex (Table 1).

### 3.2. XCA of EGC-Protein Complex

GDH catalyzes the oxidative deamination of L-glutamate and its mutations cause hyperinsulinism or hyperammonemia syndrome in animals. GTCs, especially EGC, control dysregulated GDH by hijacking the ADP activation site [113]. XCA of the GDH-EGC complex has also been discussed in a previous paper [5].

### 3.3. XCA of EGCG-Protein Complexes

Lipoxygenases are non-heme iron-containing enzymes that are ubiquitously present in nature and catalyze oxygenation of polyunsaturated fatty acids. Catechins are the natural flavonoids of known inhibitory activity toward dioxygenases that can potentially be utilized in the prevention and treatment of inflammatory diseases and cancer. XCA of soybean lipoxygenase-3 complexed with EGCG revealed an inhibitor depicting EGC that lacks the galloyl moiety (Figure 10C). Extensive hydrophobic contacts of lipoxygenase with EGC and EGCG may contribute to its strong inhibitory effect of enzymatic activity [114].

Kowalinski et al. conducted XCA of the 2009 pandemic H1N1 PA endonuclease domain with inhibitors, including EGCG and four diketo compounds, all of which can chelate the two critical manganese ions in the active site of the enzyme. Their data showed how differences in substituent groups on the basic metal binding scaffold can be orientated to bind in distinct sub-pockets within the active site cavity, and the plasticity of certain structural elements of the active site cavity, which can result in induced fit binding (Figure 10D). Their findings may be important in optimizing the design of more potent inhibitors targeting virus polymerases [118].

Heart muscle contraction is regulated by Ca^2+^ binding to the thin filament protein troponin C. In cardiovascular disease, the response of myofilaments to Ca^2+^ is often altered and compounds that restore this perturbation may be useful as therapeutics. The methods including CMDA revealed that the EGCG binding site on the C-terminal domain of troponin C lies in the hydrophobic pocket in the absence of troponin I and that binding in the presence of troponin I may be to a new site formed by the troponin C-troponin I complex. This interaction of EGCG with the C-terminal domain of troponin C-troponin I complex has not been shown with other cardiotonic molecules and may provide a potential mechanism by which EGCG modulates heart contraction [115].

Previously, EGCG was shown to bind directly to peptidyl prolyl *cis*/*trans* isomerase (Pin1) which plays a critical role in oncogenic signaling. EGCG was also reported to bind to transthyretin, which is involved in the pathogenesis of familial amyloid polyneuropathy, a disease caused by a point mutation of transthyretin, that is a thyroxine transporter. EGCG was shown to suppress amyloid fibril formation. XCA of certain features of EGCG complexes with Pin1 and transthyretin has also been described previously [5,116,117].

## 4. Characteristics of Catechin-Protein Binding Interaction Revealed by XCA

**Figure 11:**
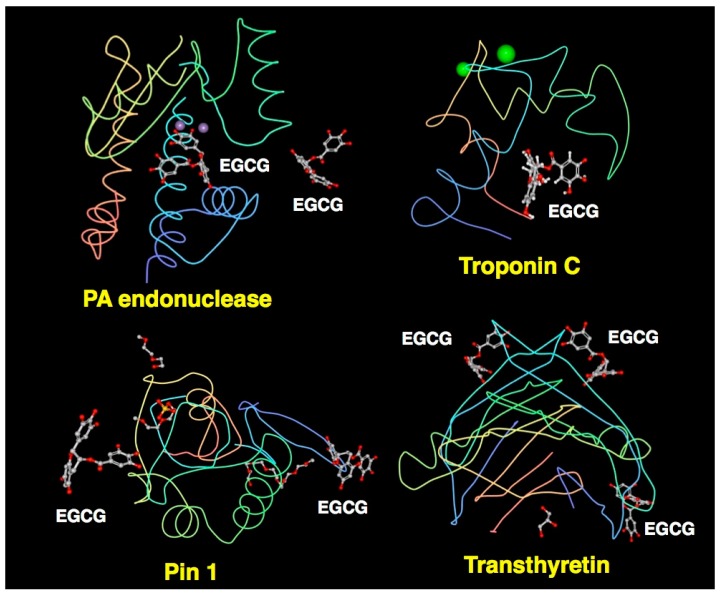
EGCG-binding to the surface of proteins. XCA of the interaction with PA endonuclease, troponin, Pin 1, and transthyretin. Figures are prepared using RCSB PDB Ligand Explorer software.

There are three common features of the catechin-protein interaction: (i) the frequent occurrence of hydrogen bonding between hydroxyl groups in the A-ring, B-ring, and the galloyl group of catechins and the side chains of Asp, Glu, and Arg; (ii) Trp and Phe can form a π-π interaction, but Tyr may rarely be involved in this interaction; (iii) the highest frequency can be found in the hydrophobic interaction with Leu, followed by His, Phe, and Ile. Among the 10 proteins examined, the highest incidence of hydrogen bonding was found in the EGCG-PA endonuclease complex (Table 1). As shown in Figure 11, EGCG is predicted to bind to the protein surface in four EGCG-protein complexes. The hydrophilic nature of the galloyl group might be involved in the interaction with water molecules. In addition, hydrophobic interactions are frequently found when EGCG interacts with branched chain amino acids (Leu/Val/Ile), His and Phe.

## 5. Conclusions

CMDA and XCA have provided detailed information about the interaction between catechins and proteins. Generally, gallate-containing catechins have higher biological activity than catechins without a galloyl group and this structure-activity can be mostly explained by CMDA and XCA which show an important role of the galloyl group in the catechin-protein molecular interaction. This is typically illustrated in the interaction with trypsin, where the galloyl group of EGCG interacts with the active cavity while the B-ring of catechin does so [14] (Figure 3). Another example may be the study on *Staphylococcal* enterotoxin A, where EGCG and EGC bind to Tyr91 in the bioactive A-6 domain, but the responsible groups differ, in that the hydroxyl group in the galloyl group in EGCG and that of the A-ring in EGC are not the same (Figure 8), providing a reason for which EGCG displays inhibitory activity against the activity of this toxin [89].

Increasing the number of galloyl groups may be one strategy for drug development. Based on CMDA, Farhan et al. proposed that the synthesis of any novel anticancer drug should have as many galloyl moieties as possible, which would provide an increased number of hydroxyl groups that can facilitate binding to a target macromolecule [32]. In addition, CMDA and XCA studies have proposed that conformational changes in protein induces further binding of EGCG to serum immunoglobulin light chains as shown in Figure 7 [32].

It should be noted that basic studies using CMDA and XCA have often been conducted on catechins without a gallate residue as described above, and future studies should include galloylated catechins. Moreover, most CMDA and XCA studies examined the protein interaction of only one type of catechins, for example, EGCG, in the absence of other catechins or related compounds. The synergistic effects between EGCG and other chemicals including catechins have often been observed. Therefore, it would also be important to examine the synergy or competition among catechins in the protein interaction by CMDA and XCA to assess, for example, how a protein interacts with EGCG in the presence of EGC.

Taken together, CMDA and XCA are effective methods to examine ligand-protein interactions and to screen lead chemical compounds. Further chemical modifications guided by structure and ligand-based computational methodologies would lead to the discovery of better clinical drug candidates.

## Figures and Tables

**Table 1:** Binding interaction between catechins/ECG analogs and protein as revealed by X-ray crystallographic analysis and solution NMR.

PDB ID	Protein	No. of Sites	Ligand	Resolution	Binding Characteristics Defined by Ligand Explorer (4.2.0)				Head Author (Year)	Reference
					No. of Hydrogen Bonds	Contact Residues and Molecules *	No. of Hydrophobic Contacts	Contact Residues and Molecules		
4MA6	Ara h 8	1	EC	2.0	7	T30(s), Y82(s), 4 waters	6	L, L, I, T, Y, H, K	Hurlburt, B. K. (2013)	[110]
3I52	Leucoanthocyanidin reductase	1	(+)-catechin	2.3	11	H122(m), Y137(s), I171(m), G93(m), E94(s), 5 waters	6	M, G, G, F, H	Mauge, C. (2010)	[111]
4C9I	PR-10 Fra a 1E	1	(+)-catechin	3.1	1 ****	D154(s)	6	L, G, H, K, D	Casanal, A. (2013)	[112]
4C94	PR-10 Fra a 3	1	(+)-catechin	3.0	5	H70(s), 4 waters	4	L, R	Casanal, A. (2013)	[112]
3QMU	Glutamate dehydrogenase	1	EGC	3.6	4 ****	V120(m), R396(s), R459(s), R491(s)	6	V, A, H, K, D	Li. C. (2011)	[5,113]
1JNQ	Lipoxygenase-3	1	EGC (EGCG) **	2.1	4	Q716(s), 2 waters	18	V, I, L, L, H, H, F, W, Q, Fe	Skrzypczak-Jankun, E. (2003)	[114]
2KDH	Troponin C	1	EGCG	n. d ***	0 ****	-	7	M, V, L, F, F	Robertson, I.M. (2009)	[115]
3NG5	Transthyretin mutant V30M	2	EGCG	1.7	5	D18(s), V20(m), 5 waters	7	V, L, I, R	Miyata, M. (2010)	[5,116]
					7	V32(m), W41(m), S46(s), E72(s), 3 waters	10	P, F, H, E		
3OOB	Pin1	2	EGCG	1.9	4	2 waters, R17(m,s)	7	R, Ligand	Urusova, D.V. (2011)	[5,117]
					5	Water R60(s), W73(m), D112(m), S114(m)	2	S, W		
4AWM	PA endonuclease	1	EGCG	2.6	12	E26(s), E80(s), D108(s), E119(s), I120(m), V122(m), K134(s), Y130(s), water	4	A, Y, H, I	Kowalinski, E. (2012)	[118]

* Words in parentheses indicate main chain or side chain atoms which participate in hydrogen bonds. ** EGC that lacks galloyl moiety binds to the substrate binding site and the iron cofactor. *** There is no resolution definition (criterion) of solution NMR. **** Water atoms were not observed because of their low resolution, their vigorous motion or solution NMR method.

## Abbreviations

3CLP	3C-like protease
AChE	acetylcholinesterase
AD	Alzheimer’s disease
BChE	butyrylcholinesterase
CDK	cyclin-dependent kinase
CMDA	computational molecular docking analysis
CYP3A4	cytochrome P-3A4
DS	Down syndrome
EC	(−)-epicatechin
ECG	(−)-epicatechin gallate
EGC	(−)-epigallocatechin
EGCG	(−)-epigallocatechin-3-*O*-gallate
GCG	gallocatechin-3-*O*-gallate
GDH	glutamate dehydrogenase
GTCs	green tea catechins
HIV-1	human immunodeficiency virus type 1
HSOS	hepatic sinusoidal obstruction syndrome
Hsp	heat shock protein
IAV	influenza A virus
LPS	lipopolysaccharide
MAPK	p38 mitogen-activated protein kinase
MCT	monocrotaline
MD2	myeloid differentiation factor 2
MetS	metabolic syndrome
MMP	matrix metalloproteinase
MRSA	multidrug-resistant *Staphylococcus aureus*
NF	nuclear factor
NGF	nerve growth factor
Nrf2	nuclear factor erythroid 2-related factor 2
OPPA	oligopeptide ABC transporter binding lipoprotein
PBD	epicatechin-3-*O*-gallate-(4β→8)-epicatechin-3′-*O*-gallate
PD	Parkinson’s disease
PDB	protein data base
Pin1	peptidyl prolyl cis/trans isomerase 1
PKC	protein kinase C
PLY	pneumolysin
PPA	pancreatic α-amylase
PPO	polyphenol oxidase
ROS	reactive oxygen species
RSK2	90-kDa ribosomal S6 kinase
SA	serum albumin
SEA	*Staphylococcal* enterotoxin A
SIRT6	sirtuin-6
SOD1	superoxide dismutase 1
SrtA	sortase A
STAT	signal transducer and activator of transcription
SULT	sulfotransferase
TNF	tumor necrosis factor
TRAF6	TNF receptor associated factor 6
TLR4	toll-like receptor 4
VEGF	vascular endothelial growth factor
